# Dynamite

**Published:** 1887-09

**Authors:** E. Van Goidtsnoven

**Affiliations:** Atlanta, Georgia


### Southern Medical Record.

A MONTHLY JOURNAL OF PRACTICAL MEDICINE.

☞All Communications must be addressed to R. C. Word, M. D., Managing Editor.

"*Quicquid Præcipies Esto Brevis.*"

Vol. XVII. ATLANTA, GA., SEPTEMBER, 1887. No. 9.

### ORIGINAL AND SELECTED ARTICLES.

### DYNAMITE!

"Monstrum horrendum et inefabile dictu!"

By E. van Goidtsnoven, M.D., Atlanta, Ga.

Dynamite is the terror of to-day and the weapon of to-morrow! There are times in the world's history when even the evolutionary forces which are constantly at work in solving social problems become effete and lack in rapidity as well as in potency in the very manifestations of their agency. Then the great soul of afflict[illegible] humanity, impatient of reform, snatches the thunderbolt and accomplishes in a day what evolution could not possibly have effected in half a century. Then it can be said that revolution rides over evolution!

Let it not be inferred that I am here vindicating nihilism or socialism. I propose to show that I am engaged in better pursuits than those of a political dreamer. But as it is in political economy, so it is in medicine. The latter, unlike mathematics, is not a positive or fixed science. Hence, it is constantly undergoing the process of evolution, until some startling discovery, such as that of chloroform, veratrum, cocaine, nitro-glycerine, breaks the spell and revolutionizes the course of investigation and practice.

Dynamite laughs at evolution! Kings and potentates tremble at its very name! But the physician hugs it *con amore* in his pocket case! Yes, to the physician nitro-glycerine is a power; not a redoutable or infernal agent, but a new medicine whose claims to be cited in our therapeutics are imperative and self-asserting!

Having read several contributions in our current medical literature claiming that nitro-glycerine exercises a powerful influence over the medulla oblongata, the functions of the pneumogastric and vaso-motor nerves, exciting the functions of the heart, increasing systemic activity, stimulating the respiratory organs, causing contraction of the cerebral arteries, and changing the rate of pulse and respiration, I determined several months ago to give it a fair test whenever an opportunity would call for its use, whether in private or hospital practice, and to report the results through the columns of the SOUTHERN MEDICAL RECORD.

CASE I. Mr. J. A. F., age 26; *acute alcoholism.* Has been drinking for a week past; cerebral anæmia well marked; cannot raise his head from the pillow; sinking sensation in the pit of the stomach and about the heart; sense of impending danger; great anxiety. "For God's sake, doctor, do something for me! I am dying!"

*Treatment.*—One drop of 1 per cent. solution of nitro-glycerine on his tongue every two hours, commencing at 1 o'clock a. m. Within five minutes after the first dose he sat on his bed complaining of intense headache and fullness around the temples. This vanished, however, in a few minutes. He continued the treatment as prescribed, and at 7 o'clock got up and went to his breakfast "perfectly cured," to use his own words!

CASE II. Aunt Phillis D., colored, ætatis 81; in *articulo mortis*; head and face cold and clammy; pulseless; stertorous breathing; lower extremities cold; thorax warm. Being called for the first time to see this patient, I inferred from the history of the case that she had been suffering with typhoid dysentery, causing a drain of the system and ending in death by anæmia. I found her dying, and so expressed it to her relatives As I was leaving the house her attending physician came in. One glance at his patient caused him to say: "She will be dead in less than an hour." Before taking my final departure, the idea struck me that possibly nitro-glycerine might rouse every vestige of latent vitality and prolong her life a few hours longer. Therefore, I let two drops of 1 per cent. solution of the drug fall on her tongue and went home. This was at 4 o'clock p. m. Judge of my astonishment when, next morning at 9 o'clock, I was informed by her delighted husband that the old lady was not only alive, but conscious and talking rationally! He begged me to go at once and see her. I started immediately, not to continue the case as she was not my patient, but to satisfy my own senses as to her true condition and determine the amount of vital energy she still possessed under the influence of that powerful stimulant. Indeed, she had rallied, nay resuscitated. Her pulse had returned. She was in full possession of her senses, spoke in plain though weak tones, and presented the appearance of a person in a very feeble and low condition. The same treatment was continued, but it soon became evident that life had exhausted all its resources, and she died at 5 o'clock p. m., twenty-four hours after taking the first dose of nitro-glycerine. I am positive that in this case life was prolonged to the extent of one day!

CASE III. Anna J., colored, ætatis 8; pulmonary c ngestion; has been sick for two weeks without any medical attendance; head and face, upper and lower extremities very cold; temperature, 95; pulseless; crepitant rale very audible; dullness on percussion; fearful sense of approaching death.

*Treatment.*—Diluted 10 drops of 1 per cent. solution of nitroglycerine in 100 drops of alcohol, and prescribed 10 drops every four hours. Improvement prompt and rapid from the first dose. On next day pulse 140, easily felt and counted; temperature $97\frac{1}{2}$; respiration easier. The same treatment was kept up for a week, at the end of which a perfect recovery was effected.

CASE IV. Miss D., age 40; Benevolent Home, Atlanta, Ga.; cancer of the bowels; extreme emaciation; cadaverous look; two pistulous openings in the abdomen, one above the pubic bone, the other in the left iliac region; fæces oozing therefrom. A more wretched picture of helplessness, misery and suffering I have never seen. Prescribed usual tonics and local applications. One evening, being informed that she was dying, I let 1 drop of 1 per cent. solution of nitro-glycerine fall on her tongue. This was immediately followed by a convulsive motion of the whole frame. In an instant she was to all appearances stiffened in death. No pulse; no heart's beat; eyes and jaws fixed. The two nurses of the Home and two inmates were present and witnessed the tragic scene. And they all exclaimed, *una et viva voce:* "Doctor, you have killed her!" I must confess I felt as though they had uttered a most solemn and awful truth, when presently I heard one exclaim: "She is coming to!" Judge of my relief! In less than three minutes the vital force was not only restored, but considerably hightened and increased. She sat on her bed, asked for a fan, became very loquacious, begged for food and tobacco, ate with appetite, asked and answered questions in a very intelligent and rational manner. Altogether it was a strange spectacle, and it looked as though Nature had entered a solemn protest against death and succeeded in reasserting her rights. This state of reanimation and exh lara-tion would last for about four hours, when all of a sudden death would come again and claim its victim. It was met and checked again and again by that terrible weapon, *dynamite*, momentarily annihilating, as it were, the poor sufferer, but restoring her almost instantly to life and renewed energy until she finally succumbed to utter exhaustion seventy-two hours after the first administration of nitro-glycerine. *Three days' grace!*

CASE V. Amanda A., colored, age 28; widow, mother of four children; syncope from menorrhagia. When I first visited this patient I found her lying on the floor in a state of suspended animation. I was informed by her friends that she had been in that condition for several hours and all attempts at reanimation had been fruitless. Was it a typical case of hysteria generally allied with disorders of uterine functions? She never had presented these symptoms before. In the eyes of some practitioners hysteria, like Charity, covers a multitude of sins, and it is very convenient sometimes, for the sinner or ignorant to hide under its cloak! Acting upon the idea that this was a case of cerebral anæmia due to drain, and it being impossible to make her swallow anything, I diluted 1 drop of 1 per cent. solution of nitro-glycerine in 10 drops of alcohol and injected it in her arm. She soon groaned and gave other evidences of returning vital force, such as raising her limbs and moving on either side. The next day she appeared to be restored to full consciousness, aphonia being the main morbid condition presenting. Prescribed the nitro-glycerine internally. On my next visit she was up and attending to her domestic duties! She has now been free from any similar attack for over a year.

CASE VI. Mrs. V.; ætatis 42; phthisis pulmonalis. I was called hurriedly to her relief by her son, who said: "Doctor, come quick, mother is dying!" Found patient propped up in bed; very pale; respiration very rapid; pulse thready; and to use Dr. Fothergills' words, "dying with pulmonary engorgement and a right heart distented nearly to paralysis." No time to be lost. Two drops of nitro-glycerine brought instant relief to the great amazement of all present, joy of the family and gratitude of the sufferer!

To conclude, I am convinced: 1. That nitro-glycerine is the most potent arm which can be placed in the hands of the physician for the purpose of combating functional diseases of the heart, from disordered enervation, characterized by mitral failure, decreased or diminished circulatory action, sinking sensation, sense of impending danger, cerebral anæmia, collapse, syncope.

2. That in many cases of grave import, digitalis, though very efficient, is too slow in its action, and should make way for the more prompt, reliable and efficacious remedy, *dynamite!* Nitroglycerine is a powerful stimulant—digitalis is a tonic.

3. That inasmuch as it has been demonstrated by indisputable authorities, such as Gaynord and McDonald, that nitro-glycerine will replace alcohol wherever and whenever the latter might be used as a cardiac and cerebral stimulant; it being also proved by the same authorities that 1 drop of the 1 per cent. solution is more than the equal of 1 ounce of brandy in such a case, it would be the part of wisdom and safe expediency to use this new therapeutic agent as a most valuable substitute. Such a course would prove a great boon in communities where *prohibition prohibits* a poor sick man from getting in a drug store what a rich sickman finds readily at home.

"*Quod licet Jovi non licet bovi.*"

